# Malignant Progression Outcomes of Bosniak Graded Renal Cysts in the Modern Era of Better Imaging and Uro-Radiology Subspecialisation

**DOI:** 10.7759/cureus.96893

**Published:** 2025-11-15

**Authors:** Francesca Speck, Abdullah Egiz, Anish Pushkaran, Farooq Khan, Barnaby Barrass, Somsundaram Ramkumar

**Affiliations:** 1 Urology, Luton and Dunstable Hospital, London, GBR; 2 Urology, Luton and Dunstable Hospital, Luton, GBR; 3 Radiology, Luton and Dunstable Hospital, Luton, GBR

**Keywords:** bosniak, bosniak cyst, malignancy risk, renal cyst, uro-radiology

## Abstract

Objective

The Bosniak grading system is used to help clinicians manage renal cysts by predicting malignant potential; however, its limitations include variable reported malignancy rates, particularly in Class IIF. Initially, these cysts were predicted to have a malignant potential of 18.5% therefore, they are followed up for at least five years. The aim of this study was to provide the outcome of Bosniak graded cysts in the modern era of CT imaging and uro-radiology subspecialisation to assess the malignant potential of each class.

Methods

A total of 379 Bosniak graded cysts were analyzed over a five-year period, with a median follow-up of 11.2 months. Malignant potential was calculated based on radiological progression or histopathology.

Results

Subsequent malignant transformation rates were 0% in category 1, 1.70% in category 2, 3.96% in category 2F, 27.78% in category 3, and 61.54% in category 4.

Conclusion

This study adds to the growing body of evidence that Bosniak cysts, especially Class IIFs, are less malignant than previously thought. Stringent evaluation of these cysts in dedicated renal multidisciplinary teams (MDTs) is to be encouraged along with further research. This will contribute to a refinement of the follow-up guidelines to ensure they are both appropriate and safe.

## Introduction

Renal cysts are present in 5% of the general population, with prevalence increasing to 25% in individuals older than 50 years [1]. Most of these are benign incidental findings in asymptomatic patients; however, some have the potential to transform into malignant lesions. In order to better classify these lesions and avoid the need for invasive surgery, the Bosniak classification system is utilised. The Bosniak classification system has been widely adopted, and its accuracy in ruling out malignancy has been confirmed [2,3]. However, over the years, limitations to its use have been noted, specifically significant inter-reader variability and, of particular interest in this study, controversy amongst malignancy rates. The malignant transformation rates were originally quoted in each cyst class by Warren and McFarlane in their 2005 paper. As a result, contemporary guidelines suggest that class I and II do not require follow-up; however, IIFs require follow-up for five years by annual CT scans. Class III/IV are generally treated if the patient is an appropriate candidate for intervention [4,5].

Over the years, the class IIF has gained attention as a source of controversy due to the variability in malignancy rates in the literature. Some studies have noticed that IIF cysts have a lower rate of malignant potential than previously thought, with them also being overdiagnosed in a significant proportion of people [6-13]. However, controversy exists as other studies suggest a higher malignant potential of IIF cysts, demonstrating a need for further research [14-16]. Therefore, the purpose of this study was to look at the malignant conversion rates of Bosniak cysts in the modern era of CT imaging in our centre, where cysts underwent uro-radiology multidisciplinary team (MDT) review. Hopefully, studies such as this contribute to a refinement of the follow-up guidelines to ensure they are appropriate and safe.

## Materials and methods

This was a retrospective single-centre study in which all images from a five-year period with Bosniak grading were collated by our radiologist. The time period analysed was 2017-2022, which included 511 total scans. All duplicates were removed from the data. The data was collected by 2 resident doctors at the trust working within surgery. All five categories of Bosniak graded cysts were included in the analysis (I, II, IIF, III, IV), and we only excluded imaging reports that reported simple cysts without an official grading. We also excluded those who were found to have pathophysiology other than a renal cyst, such as tumours or cancers from other origins. This search did not exclude any form of imaging, so ultrasound, CT, and MRI were all included. We obtained approval from our institution's audit department to conduct the study; however, written informed consent was not required due to the retrospective nature of the data collection.

Each patient record was then reviewed in more detail to identify the date of initial diagnosis of the cyst and how it had progressed over time until either follow-up ceased or the most recent scan was reached. Although the primary study period covered 2017-2022 (five years), some patients had undergone earlier imaging prior to this interval. To accurately capture the complete disease course for these individuals, data were extended to include these earlier scans, resulting in a total review period of up to seven years. If a patient’s scan went to MDT, the Bosniak classification given at this meeting, where urologists and uro-radiologists were present, was documented in the data as the initial grade of the cyst (even if this varied from the initial scan report).

The finalised data set was analysed using Microsoft Excel. Descriptive statistical analysis was performed to summarise patient demographics, imaging characteristics, and cyst outcomes. Categorical data were expressed as absolute numbers and percentages, while continuous variables were summarised using means and ranges where appropriate. The primary analytical outcome was the proportion of Bosniak cysts within each category that were upgraded to a higher classification or demonstrated malignant transformation, which was used to determine the progression rate across the cohort. Confidence intervals were not calculated, as the study was designed to provide a comprehensive descriptive overview of all identified cases rather than to perform inferential or comparative statistical testing.

## Results

**Figure 1:**
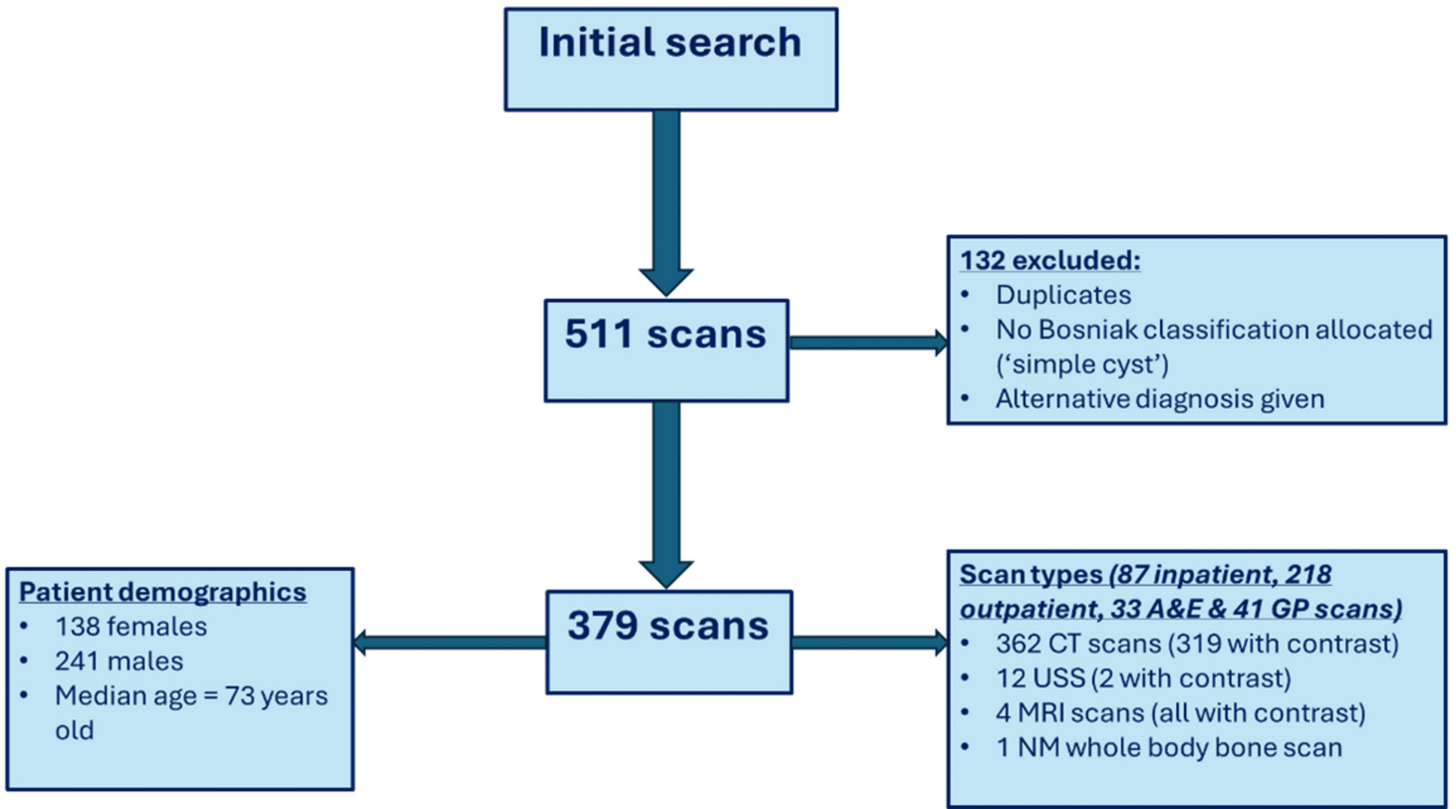
Flowchart of scan selection process with exclusion criteria A&E: Accident and emergency, GP: General practitioner, USS: Ultrasound scan, NM: Nuclear medicine.

The final sample included 379 Bosniak graded cysts after exclusions were applied to the dataset, as shown in Figure 1 (flowchart of scan selection process with exclusion criteria).

The final data set included 138 (36.4%) females and 241 (63.6%) males with a median age of 73 years old (Standard Deviation (SD) = 15.6). Out of the scans performed, 87 (23.0%) were inpatient, 218 (57.5%) were outpatient, 33 (8.7%) were done in the Accident and Emergency (A&E), while 41 (10.8%) were via the patients' general practitioner (GP) surgery. Out of the total, 362 (95.5%) of the total scans were CT scans, with 319 (88.1%) of these performed with contrast. However, 12 (3.2%) of the scans were USS (two, 18.2%) with contrast, four (1.1%) were MRI with contrast, and one (0.3%) was nuclear medicine (NM) whole body bone scans.

The CT scans took various forms as demonstrated in Table 1. Two of the patients had two different types of scans at the same time, increasing the total number to 364 instead of 362. One patient had a CT renal with angiogram, whilst the other had a CT thorax, abdomen, and pelvis with angiogram. The most commonly performed scan was a variation of a CT scan of the neck, abdomen, pelvis, and/or thorax, accounting for almost half of the scans at 167 (45.9%). This was further broken down into individual scans of the abdomen, pelvis, and thorax, or a combination of them. This was followed by CT scans of the urinary tract in 87 (23.9%) and then of the kidneys in 76 cases (20.9%). 

**Table 1:** CT Variations

CT scan	Number performed
Urinary tract	87 (23.90%)
Kidneys	76 (20.90%)
Neck/thorax/abdomen/pelvis	167 (45.88%)
Angiogram	13 (3.57%)
Trauma	5 (1.37%)
Skeletal	1 (0.27%)
Cervical spine	5 (1.37%)
Contrast enema	2 (0.55%)
Other organ-specifc	8 (2.20%)
Total	364

The numbers of each classification of Bosniak cysts are demonstrated in Table 2 along with their respective malignant transformation rates. The Class II Bosniak classification made up almost half of the cysts analysed, at 176 (46.4%), with class IIF following, making up 101 (26.6%) of the total cysts. There were 71 (18.7%) class I cysts, 18 (4.7%) class III, and lastly 13 (3.4%) class IV cysts. None of the class I cysts were upgraded or transformed into malignancy in the follow-up period, whereas only three of the 176 class II cysts underwent this fate. Additionally, four of the 101 class IIF cysts progressed in the time period analysed. Finally, five of the 18 class III cysts and eight of the 13 class IVs underwent malignant progression.

**Table 2:** Bosniak cysts with their associated malignant progression rates

Bosniak classification	Number of cysts (%)	Malignant progression rate (%)
I	71 (18.7)	0
II	176 (46.4)	3 (1.70)
IIF	101 (26.6)	4 (3.96)
III	18 (4.7)	5 (27.78)
IV	13 (3.4)	8 (61.54)
	Total: 379	

One hundred and thirty-seven (36.15%) of the cysts were referred to MDT for discussion. Seven (5.11%) of these were class I, 42 (30.66%) were class II, 59 (43.07%) were class IIF, 16 (11.68%) were class III, and 13 were class IV (9.49%). Table 3 shows the percentage of each Bosniak cyst classification that went to MDT and were subsequently followed up.

**Table 3:** Amount of Bosniak cysts that underwent MDT discussion MDT: Multidisciplinary team.

Bosniak classification	MDT discussion took place (%)	MDT outcome
I	7 (9.86)	No further urology input: 4
		Follow-up: 2 (2 followed up who did not go to MDT)
		Lost to follow up: 1
		Intervention: 0
		Total followed up: 4
II	42 (23.86)	No further urology input: 19
		Follow-up: 20 (8 followed up who did not go to MDT)
		Lost to follow up: 0
		Intervention: 3 (both had follow up imaging also)
		Total followed up: 31
IIF	59 (58.42)	No further urology input: 10
		Follow-up: 41 (11 followed up who did not go to MDT)
		Lost to follow up: 4
		Intervention: 5 (2 had no follow up imaging prior/after)
		Total followed up: 55
III	16 (88.89)	No further urology input: 2
		Follow up: 9 (2 followed up who did not go to MDT)
		Lost to follow up: 1
		Intervention: 4 (after follow-up)
		Total followed up: 15
IV	13 (100)	No further urology input: 4
		Follow-up: 1
		Lost to follow up: 1
		Intervention: 7 (1 went straight to intervention and had no follow up imaging prior/after)
		Total followed up: 7

The median follow-up duration for all of the cysts combined was 11.2 months. Table 4 shows the breakdown of the number and length of follow-up that took place for each individual classification. Class IIF cysts were followed up for a median length of 14 months (SD = 12.7), but the range was 61 months (5.1 years). Out of all the cysts, the class III’s were the most followed-up category and also had the longest median length of follow-up.

**Table 4:** Follow up of Bosniak graded cysts Note: The percentages in this table are demonstrating the amount of cysts within each class that were followed up.

Bosniak classification	Followed up (%)	Median length in months
I	4 (5.63)	1
II	31 (17.61)	9
IIF	55 (54.46)	14
III	15 (83.33)	21
IV	7 (53.8)	11
	Total followed up: 110 (29.02%)	Total median follow up in months: 11.2

**Figure 2:**
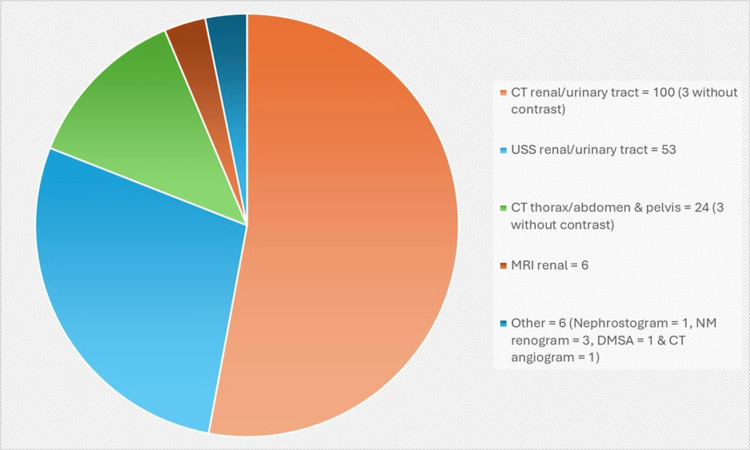
Summary of follow-up scans performed

The breakdown of the follow-up scans performed is summarised in Figure 2. The majority of follow-up scans took the form of CT scans of either the kidneys or the urinary tract, making up 100 (52.91%). This was followed by ultrasound scans (USS) of the urinary tract, which accounted for 53 (28.04%) of total follow-up images. Twenty-four (12.70%) were CT scans of either the thorax, abdomen, pelvis, or a combination of these. Six (3.17%) were contrast-enhanced MRI scans, while there were three (1.59%) NM renograms and one (0.53%) nephrostogram, dimercaptosuccinic acid (DMSA) scans, and CT angiograms. Ninety-seven (95.16%) of the CT scans were performed with contrast, along with six (100%) of the MRIs. None of the ultrasounds were contrast-enhanced in this study.

Out of all the 379 cysts analysed, only 17 (4.75%) had either an operation or intervention for their cyst: three of the Bosniak II cysts, five of the IIFs, three of the IIIs, and six of the IVs. None of the Bosniak class I cysts underwent intervention. A summary of the intervention performed, the time it took in months before this took place, and the histology (when available) is summarised in Table 5. There were 12 nephrectomies performed in total, six of which were partial and four that were radical. Other procedures performed included deroofing or drainage of the cyst, and also cryotherapy. One patient received chemotherapy, where surgery was not suitable. The most common histology finding was of renal cell carcinoma (RCC), which was found in 10 cases (55.56%). Most of the RCCs were found in the class IV cyst resections, but there was also one found in each class II, IIF, and III.

**Table 5:** Surgical interventions performed PN: Partial nephrectomy, RN: Radical nephrectomy, RCC: Renal cell carcinoma.

Bosniak classification	Number that required intervention (%)	Intervention	Time to intervention (months)	Histology
I	0	N/A	N/A	N/A
II	3 (1.70)	Nephrostomy + drainage of cyst	3	N/A
		Open RN	34	RCC
		Laparoscopic RN	3	Papillary RCC
IIF	5 (4.95)	Cryotherapy	23	Oncocytoma
		PN	5	Type I papillary carcinoma and papillary adenoma
		PN	5	Oncocytic renal cell neoplasm
		PN	7	Clear cell RCC
		Laparoscopic de-roofing of cyst	8	Simple renal cyst
III	3 (16.67)	Cryotherapy	18	Clear cell RCC
		PN	8	Cystic RCC
		Robotic PN	4	Oncocytoma
IV	6 (46.15)	Nephrectomy	2	Chromophobe RCC
		PN	3	Clear cell cystic RCC
		RN	2	Chromophobe RCC
		RN	3	Chromophobe RCC
		Laparoscopic nephrectomy	2	Clear cell RCC
		Chemotherapy	2	RCC

## Discussion

The most interesting finding from this study was the lower malignant transformation rate in all Bosniak classes when compared to historical data. Of particular interest is the 3.96% malignant potential of class IIF. This malignant transformation figure is similar to that found in four other recent studies by Shen et al. in 2023 [10], which found a rate of 4%, Lucocq et al. in 2020 [12], and Tames et al. in 2019 [13], who both found a malignancy rate of 4.6%. Shen et al. (2023) [10] also reported a similar median age of 72 years old, and both this article and Lucocq et al.'s (2020) [12] reported a similar distribution of males to females, with men making up between 58 and 67%. All papers defined malignancy in the same way as this paper by looking at the percentage of cysts re-classified to a higher grade. This definition was utilised because the literature suggests that reclassifying a Bosniak IIF cyst to a III/IV is highly indicative of malignancy [7]. The other studies reported lower malignancy rates than these ones, ranging from 0.3 to 2.2% [6-11]. Despite this growing body of evidence, there is some controversy; for example, one study reported the progression rate as high as 60%. The main difference was that this study relied on pathological diagnosis of malignancy [16]. However, it has been suggested that reviewing histopathology alone may overestimate malignancy rates [7]. This is because the rate of malignancy amongst surgically resected cysts is high, but there is still a high proportion of benign cyst resections [17]. Additionally, this study focused on a younger age group with the median age being 59 years old, perhaps giving the cysts more time to transform as one proposed theory [16]. 

It is well recognised that the Bosniak classification has some limitations, which partly explain the variation in reported malignancy rates of IIF cysts. Most notable is the subjective nature of the classification, resulting in high variability in the interobserver agreement rate, increasing the risk of incorrect diagnosis, follow-up, and management [18-21]. Although it is suggested that uro-radiologists who routinely evaluate complex renal cysts can utilise the classification in a reproducible way [21]. 

Due to these complexities, it is undetermined exactly how these cysts should be followed up. It is generally recommended that Bosniak IIF cysts are followed up six-monthly for one year and then annually for five years, but there are no specific UK guidelines to follow [22]. This was based on a malignancy rate of ~15% but if, in fact, the actual progression is lower than this, perhaps the follow-up guidelines should be refined. The follow-up of Bosniak IIF cysts has been noted to be a significant financial and workload burden despite low intervention rates. Therefore, it has been suggested that MDTs could be implemented to determine individualised follow-up regimens [23]. Not to mention, the repetitive radiation exposure to the patient, stress, and anxiety that may also be caused [6]. One study suggested that follow-up could allow for larger intervals between scans to allow cysts to progress, but also reduce radiological exposure [17]. This demonstrates the importance of understanding the true malignancy rates of Bosniak cysts so we can ensure follow-up is current, specific, and appropriate.

Limitations

The study design was flawed in that we reviewed images over a period of five years, meaning that each patient had a varied length of follow-up from the initial scan to the present day. However, this is difficult to carry out in reality due to them still being relatively uncommon at 5% in the general population; therefore, data needs to be collected over a period of years to get a significant sample size [1]. Although it would appear that most cysts will progress in the first two years, with progression being rare after five years of follow-up [24]. Additionally, there was a significant amount of class IIF Bosniak cysts that were not followed up on. The main reasons for this were patients not attending appointments, patient death, and the rest were for unknown reasons.

Lastly, in this study, the updated 2019 classification was not utilised as CT images were performed in the majority. This update was introduced in order to improve the classification, increase the accuracy of predicting malignancy, and reduce the high interreader variability [3]. However, it has been suggested that the increased complexity of the new classification is an issue; therefore, interreader agreement and specificity have not been improved significantly [25]. In addition, the paper from Shen et al. in 2023 utilised the 2019 classification, and this resulted in a similar progression rate of 4% [10].

The future

Various papers have begun to explore more creative methods for detecting potentially malignant cysts using advanced technologies, such as machine learning algorithms, which have demonstrated success in reducing interreader variability [26]. Furthermore, proposed novel scoring systems such as the renal cyst index (RCI) have been demonstrated to outperform the Bosniak classification, especially when evaluating IIF and III cysts for malignancy [27]. Other solutions may include exploring alternative methods to reduce radiation exposure, such as using contrast-enhanced ultrasound [28]. Image-guided biopsies could also be considered more frequently to avoid over-treatment of benign lesions [29].

## Conclusions

To summarise, strict evaluation of Bosniak cysts of class IIF and above in dedicated renal MDTs where a specialist uro-radiologist is present is recommended to improve the accuracy of the diagnosis, inter-reader variability, and provide the most beneficial outcome for the patient. More research and better techniques are needed in this area to redefine the follow-up criteria in a way that minimizes harm and unnecessary intervention in patients with Bosniak cysts. However, the results from this study add to the body of evidence that the malignancy rates of IIF and III cysts are lower than expected, providing an argument for reducing the follow-up period or increasing the intervals between follow-up scans for this purpose.
